# Surgical treatment of patent ductus arteriosus in different age groups

**DOI:** 10.1590/1677-5449.202301132

**Published:** 2026-03-23

**Authors:** Abdurrahim Colak, Necip Becit, Ugur Kaya, Munacettin Ceviz

**Affiliations:** 1 Ataturk University, Faculty of Medicine, Department of Cardiovascular Surgery, Erzurum, Turkey.; 2 Afyonkarahisar Healt Sciences University, Department of Cardiovascular Surgery, Afyonkarahisar, Turkey.

**Keywords:** PDA, ligation, division, PCA, ligadura, divisão

## Abstract

**Background:**

Patent ductus arteriosus (PDA) is the continuation into postnatal life of a normal fetal vascular structure that makes an anatomical connection between the central pulmonary arterial system and the systemic arterial system.

**Objectives:**

Patent ductus arteriosis (PDA) is a normal fetal vascular structure that makes a connection between the central pulmonary arterial system and the systemic arterial system, which is the continuation of the anatomical connection in postnatal life. In this study, we aimed to evaluate the results of PDA in our clinic with current literature.

**Methods:**

In this study, data were reviewed retrospectively from 159 cases operated from 2000 to 2018 in the Department of Cardiovascular Surgery after diagnosis of PDA. Patients’ ages ranged from 3 days to 47 years and mean age was 5.48 (± 0.70) years. Seventy (44%) of these patients were female and 89 (56%) were male.

**Results:**

The most common reason for admission of the patients included in the study was diagnosis at another health institution that referred them to our clinic. PDA interruption was achieved in 3 of them with a division technique. None of our patients entered total cardiopulmonary arrest. A ligation technique was used in 121 (76.1%) of the patients who underwent PDA closure with posterolateral thoracotomy. Seventy-five (47.1%) of the patients who underwent ligation were treated with a silk ligation technique; hemoclips and PDA elimination were employed in 21 (21.2%); and hemoclips and silk ligation were employed together in 25 patients (15.7%).

**Conclusions:**

A surgical approach with thoracotomy for PDA closure is a treatment that can be employed successfully in children and adults as well as in older patients.

## Resumo

**Contexto:**

A permeabilidade do canal arterial (PCA) é uma estrutura vascular fetal normal que liga o sistema arterial pulmonar central e o sistema arterial sistêmico, que é a continuação da conexão anatômica na vida pós-natal.

**Objetivos:**

A persistência do canal arterial (PCA) é uma estrutura vascular fetal normal que estabelece uma conexão entre o sistema arterial pulmonar central e o sistema arterial sistêmico, sendo essa conexão anatômica uma continuidade na vida pós-natal. Neste estudo, objetivamos avaliar os resultados da PCA em nossa clínica, comparando-os com a literatura atual.

**Métodos:**

Neste estudo, foram analisados retrospectivamente os dados de 159 casos de pacientes operados com o diagnóstico de PCA no Departamento de Cirurgia Cardiovascular entre 2000 e 2018. As idades dos pacientes variaram entre 3 dias e 47 anos e a média de idade era de 5,48 (± 0,70). Dos casos, 70 (44%) eram do sexo feminino e 89 (56%) do sexo masculino.

**Resultados:**

O motivo mais comum de apelo dos pacientes incluídos no estudo era o resultado do diagnóstico de outra instituição de saúde e o encaminhamento para nossa clínica. Em três deles, obteve-se o fechamento da PCA pela técnica de divisão. Nenhum de nossos pacientes entrou em parada cardiorrespiratória total. A técnica de ligadura foi usada em 121 (76,1%) dos pacientes que foram submetidos ao fechamento da PCA com toracotomia posterolateral. Nos pacientes em que foi aplicada a técnica de ligadura, aplicou-se a técnica de ligadura de seda em 75 (47,1%), foram utilizados hemoclipes e fechamento da PCA em 21 (21,2%) e em 25 (15,7%) foram utilizados tanto hemoclipes quanto ligadura de seda.

**Conclusões:**

A abordagem cirúrgica com toracotomia no fechamento da PCA é um tratamento que pode ser aplicado com sucesso em crianças, adultos e idosos.

## INTRODUCTION

The ductus arteriosus is an arterial vascular structure located between the proximal part of the left pulmonary artery and the aortic arch, typically near the origin of the left subclavian artery. In cases of an aberrant origin, its anatomy may vary. Rarely, it may originate from the aorta or branches other than the aorta (ascending aorta, brachiocephalic artery, carotid or subclavian arteries), and merge to the main and right pulmonary artery, in which case it can be termed atypical PDA.^1^ In newborns born at normal time and without low birth weight physiological closure occurs within the first 12 hours after birth. This is followed by anatomic closure that takes place within 2-3 weeks. The ductus tissue turns into a fibrous residue after anatomical closure and is called the ligamentous arteriosus.^2^ The incidence of isolated PDA in full term infants is 12 in 2000 live births. It constitutes 5-10% of all congenital heart diseases.^3^ It is 2 times more common in girls than in boys.^4^

Since the pulmonary and systemic vascular resistances are similar to each other immediately after birth, the magnitude of shunt is very low, regardless of the size of the PDA.^5^ If pulmonary hypertension develops in the future, increased pulmonary vascular resistance decreases blood flow again.^2^ Increased pulmonary blood flow creates extra volume load. While this blood flow does not cause a significant hemodynamic change in small and medium PDAs, it may progress to pulmonary hypertension and subsequent right heart failure in large PDAs with shunts.

Our aim in this article is to reevaluate in the light of current publications the results of patients of different age groups who we treated surgically.

## MATERIAL AND METHODS

Between January 2000 and December 2020, the treatment results of 159 patients who were followed-up and treated with congenital cardiac pathologies accompanied by PDA were evaluated at the Department of Cardiovascular Surgery. All patients with early and late follow-up who underwent surgical treatment after diagnosis of PDA were included in the study. Patients who had PDA closure with a catheter applied to the PDA and patients who underwent medical therapy for PDA closure outside of our clinic were not included in the study. Surgical methods employed in the patients, post-treatment complication rates, postoperative hospitalization duration, whether there was any effect on cardiac functions and if so in which direction, and treatment mortality were all examined. Neonatal patients diagnosed with PDA and hospitalized in pediatric intensive care were followed up in the intensive care unit after surgery. Patients with primary diagnosis of PDA who were hospitalized in our clinic due to PDA were included in the study.

**Figure 1:**
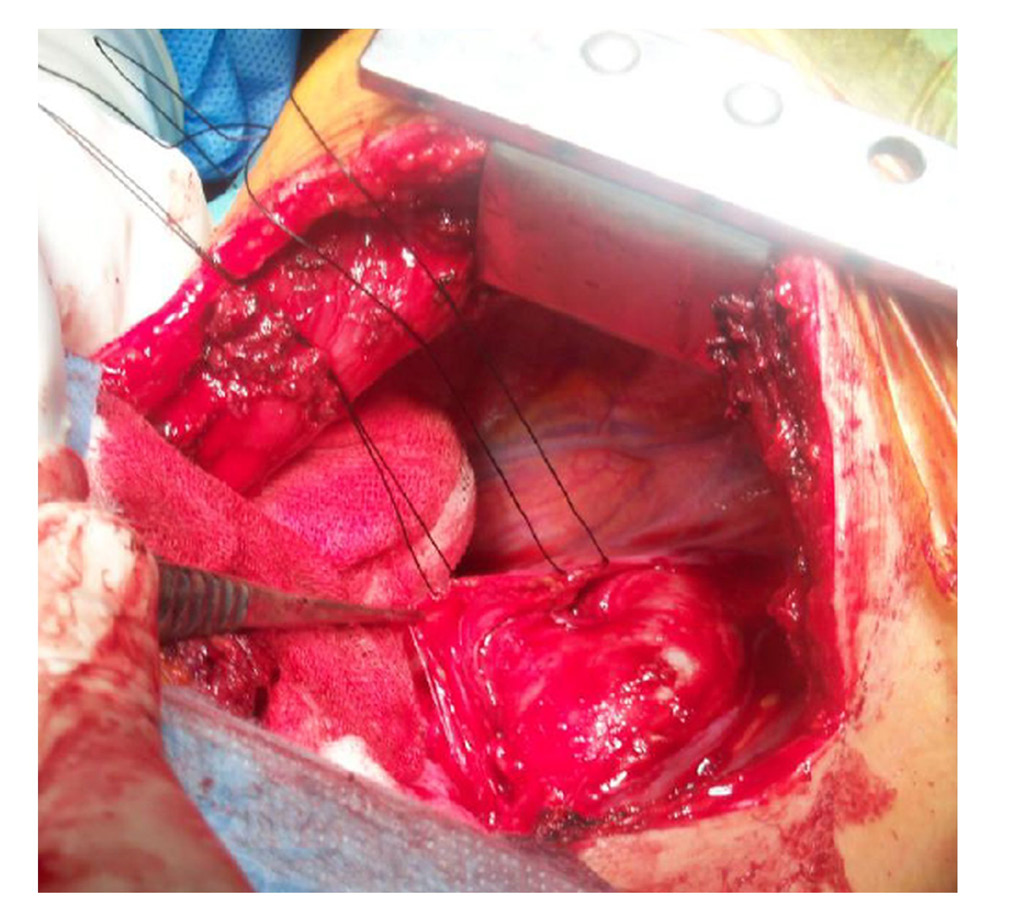
Intraoperative view of PDA.

Patients with no disability preventing the operation were treated with surgery. They were monitored and intubated under general anesthesia. Invasive arterial pressure measurements were performed, except for those who would be treated for neonatal and pediatric PDA surgery only. Patients undergoing surgical treatment with thoracotomy were placed in the left lateral decubitus position and the PDA was reached by posterolateral thoracotomy (Figure 1). Invasive arterial pressure measurement and central venous pressure measurement were performed for patients who were going to undergo combined surgical treatment due to concomitant cardiac anomalies. Patients who were deemed suitable for supine position surgery underwent median sternotomy. In the left posterolateral approach, the ductus was reached with the extra pericardial method (Figure 1).

Our article complies with the Declaration of Helsinki and local ethical guidelines and was approved at the Atatürk University Faculty of Medicine Department of Cardiovascular Surgery meeting number B.30.2.ATA.0.01.00 / 40, dated 24.05.2012, and meeting number B.30.2.ATA.0.01.00 / 40.

### Statistical evaluation

SPSS 21 software for Windows was used to evaluate the findings obtained in our study as the procedures were not equal in all age groups. While evaluating the study findings, data were subjected to analysis of variance within the scope of the General Linear Model for comparison of descriptive statistical methods (mean, standard deviation) as well as for comparisons between groups having normal distribution parameters in the general data. Significance was evaluated at the level of p <0.01.

## FINDINGS AND RESULTS

PDA closure was performed on a total of 159 cases who underwent surgical treatment. Seventy (44%) of these patients were female and 89 (56%) were male. The ages of PDA patients on whom we performed the surgical treatment varied from 3 days to 47 years and the average age was 5.48 (± 0.70) years. 48 of the cases (30.2%) were in the 0-1 month group (neonates); 51 cases (32.1%) were in the 1-12 months group (infants); 50 cases (31.8%) were in the 1-18 years group; and 10 cases (6.2%) were in the >18 years age group. 28.3% (45 cases) of the patients who underwent surgery for PDA in our hospital were asymptomatic (Table 1). The pulmonary artery pressure of the patients included in this study group was measured before the operation, 1 month after the operation, and 6 months after the operation, and the change in pulmonary artery pressure after occlusion of the ductus arteriosus was analyzed.

**Table 1:** Reasons for hospital admission of the operated patients.

**Patients**	**(N)**	**%**
Asymptomatic	45	28.3
Recurrent lung infections	30	18.8
Respiratory distress syndrome	20	13
Effort dyspnea	16	10
Growth retardation	16	10
Tachycardia	10	6.2
Tachycardia + growth retardation	5	3.1
Cyanosis	4	2.5
Tachycardia + cyanosis	7	4.4
Effort dyspnea + growth retardation	3	2.3
Effort dyspnea + cyanosis	5	3.1

PDA was closed in adult patients who had not developed Eisenmenger syndrome and in patients with suitable pulmonary pressures. Pulmonary artery pressure of patients included in this study group was measured before the operation, 1 month after the operation, and 6 months after the operation, and the change of pulmonary artery pressure after elimination of the ductus arteriosus was analyzed. Pulmonary artery pressure started to decrease after the operation and followed a decreasing trend at the 1st and 6th months in our PDA patients without any other accompanying cardiac pathologies. Statistical analysis showed that pulmonary artery pressure was significantly reduced after PDA elimination (p <0.01). The most common accompanying cardiac pathology was ASD in 40 patients (25.1%) (Table 2). When the correlation between ductus diameters and preoperative pulmonary artery pressure was observed, it was found that pulmonary artery pressure increased significantly as ductus diameter increased (p <0.01).

**Table 2:** Accompanying anomalies.

**Concomitant anomalies**	**Patients(n)**	**%**
Atrial septal defect (ASD)	40	25.1
ASD + ventricular septal defect (VSD)	13	8.1
VSD	7	4.4
Coarctation	5	3.1
Coarctation + VSD	6	3.7
Coarctation + ASD	11	6.9

Patients operated due to diagnosis of PDA were evaluated with echocardiogram before the operation, 1 month after the operation, and 6 months after the operation and the effects of PDA elimination on the right and left atrium diameters were examined. The right and left atrium diameters had decreased at the echocardiogram evaluations conducted 1 month and 6 months after the operation and this decrease was statistically significant in the pediatric group under the age of 5 (p <0.01).

147 of the surgical procedures performed on our patients (96.0%) were performed with a left posterolateral thoracotomy and there was no need for cardiopulmonary bypass. PDA closure under cardiopulmonary bypass was performed by median sternotomy in 12 patients (3.4%) to repair accompanying cardiac pathologies and 9 of these 12 patients were treated using a silk ligation technique. PDA elimination was achieved in 3 of them with a division technique. None of our patients entered total cardiopulmonary arrest. A ligation technique was used in 121 (76.1%) of the patients who underwent PDA closure with posterolateral thoracotomy. Among the patients to whom a ligation technique was applied, 75 (47.1%) were treated with a silk ligation technique, hemoclips and PDA elimination were employed in 21 (21.2%), and hemoclips and silk ligation were applied together in 25 (15.7%). End-to-end anastomosis of the aortic segments was performed concurrently for aortic coarctation in 13 (10.2%) of 128 patients who underwent PDA closure via posterolateral thoracotomy. In 17 patients (10.6%) who underwent PDA closure with left thoracotomy, PDA elimination was achieved by a division technique. In one of the 12 patients who underwent cardiopulmonary bypass surgery with a heart lung pump, an Amplatzer device fell into the right ventricular cavity and inserted into the tricuspid leaflets during the PDA elimination operation using the catheter method. This patient was taken for an emergency operation, PDA ligation was performed under cardiopulmonary bypass with the median sternotomy method, and the Amplatzer device was removed with a right atriotomy (Table 3).

**Table 3:** Surgical methods.

**Surgical methods**	**Patients(n)**	**%**
Silk ligation	75	47.1
Clips	21	13.2
Ligation + clips	25	15.7
Division	17	10.6
Division + coarctation resection	13	8.1
With CPB, silk ligation and division	12	7.5

CPB: cardiopulmonary bypass.

The shortest postoperative hospitalization period among our patients was 3 days, and the patient who remained in hospital the longest stayed for 23 days, with an average postoperative stay of 5 days. Upon reviewing postoperative complications, we found that 4 patients who had undergone PDA ligation required repeat closure between 2 and 6 months after discharge. Coil embolization was subsequently performed by the pediatric cardiology clinic in all 4 cases. One patient was hospitalized in our clinic after detection of residual PDA during routine outpatient control 1 year after the operation and the PDA was then eliminated with a division method. In 1 patient, a left thoracic hematoma was observed 8 hours postoperatively. No bleeding focus was detected in the operation; hematoma drainage was provided. None of our patients had infection, aneurysm formation, vagus nerve or laryngeal nerve injury, or chylothorax.

The total mortality of the PDA operations performed in our clinic was calculated as 8 patients (5%) (Table 4). All (newborn) patients who underwent a ligation technique with left posterolateral thoracotomy had mechanical ventilator follow-up on the 1st postoperative day, and one of them subjected to mechanical ventilator complications died on the 5th postoperative day due to aspiration pneumonia.

**Table 4:** Operative mortality.

**PDA Mortality**	**Surgical method**	**Operative mortality**
Neonates (0-1 month) (n=48)	Division 2	5.2%
	Ligation 6	4.9%
Infants (1 month-12 months) (n=51)	Division 0	
	Ligation 0	
Children (1 year to 18 years) (n=50)	Division 0	
	Ligation 0	
Adults (>18 years old ) (n=10)	Division 0	0%
	Ligation 0	0%

## DISCUSSION

Patent ductus arteriosus (PDA) is the continuation into postnatal life of a normal fetal vascular structure that makes an anatomical connection between the central pulmonary arterial system and the systemic arterial system.^1^ PDA is the most common type of abnormal relationship between large vessels.^6^ PDA accounts for 5-10% of all congenital heart diseases (CHD). It can be associated with various other CHD. The prevalence in adulthood is 0.05% and it is usually an isolated lesion.^7^

PDAs in adults constitute a special cardiac condition. The impact of long-standing PDA-related shunt on adults who probably have acquired cardiomyopathies or systemic illness is different from the impact in the pediatric population. The annual mortality rate is 1.8% in untreated adult PDA patients (without correction according to PDA diameter).^8^ Detailed studies by Campell also revealed that 34% of patients with PDA died before the age of 40 and 61% died before the age of 60 if they were not operated on.^9^,^10^

Depending on the ductus diameter of patients with PDA, on the relationship between pulmonary and vascular resistance, and on the strength of the myocardium that works against increased load, there can be a wide range of clinical presentations, ranging from asymptomatic to severe heart failure. In a study with 117 patients conducted by Russell G. Fisher et al., the reasons for admission to hospital were asymptomatic in 37 patients (32%), exercise intolerance in 37 patients (32%), and cyanosis in 9 patients (7.6%).^11^ In our study, the most common reason for patients to be admitted to the hospital was asymptomatic, in 43 patients (34.13%); 20 patients (15.87%) had recurrent respiratory infections; 17 patients (13.49%) were hospitalized for respiratory distress syndrome (RDS); exercise dyspnea was found in 11 patients (8.74%); developmental retardation in 10 patients (7.93%); and palpitations in 6 patients (4.77%) (Table 1). In this study, if we consider that PDA is associated with a very wide clinical picture, we note that admissions to the hospital were made with a similar clinical picture to the studies exemplified above.

The machine-gun style murmur PDA described by Gibson is a characteristic finding of PDA and is often sufficient to make a diagnosis. Apart from this, hyperactive pericardium, increased left ventricular activity, and thrill found along the upper left edge of the sternum are noteworthy.^11^ In patients with small shunts, chest radiography may be completely normal. Radiologically, the enlargement of the heart shadow draws attention, especially the left atrium and left ventricle. The main pulmonary artery segment is enlarged significantly and an increase in lung vascular shadows occurs. As in other left-right shunt cases, pulmonary vascularity is observed to be increased more on the right side.^12^,^13^ In the study by Fisher et al.,^11^ 114 of 117 patients were found to have systolic murmurs ranging from second to fourth degree. Continuous murmur was detected in 67% of patients. No murmur was heard in 4 patients. It was found that 23% of patients had right ventricular hypertrophy and 15.8% had left ventricular hypertrophy findings.^11^ In our study, isolated PDA murmur was the most common physical examination finding, found in 64 patients (50.7%), while 23 patients (18.3%) had PDA murmur as well as hepatomegaly, 20 patients (15.8%) had thrill accompanying PDA murmur. Since there was aortic coarctation accompanying PDA, there was absence of femoral pulse beside murmur, presence of thrill, and hepatomegaly in 7 patients (5.6%) with classical murmur, and also hepatomegaly and jugular venous filling without any cardiac murmur accompanied by signs of right heart failure were detected in 4 cases.

Echocardiography is becoming more and more widespread in diagnosis of congenital heart diseases and enables more trustworthy data to be obtained. This method is very reliable for diagnosing PDA.^14^ Echocardiogram, CT was taken with catheterization and Echocardiogram was considered to be the main diagnostic criterion. In the preoperative investigations of our patients, pulmonary hypertension (PAB> 40 mmHg) was detected in 58 (46.0%) of 126 patients and similar results to the literature were found.

In recent years, the number of surgical interventions for PDA has increased in premature babies, especially at advanced centers. However, the reason for this increase is due to the increase in the number of smaller premature babies. According to the statistics for the last 10 years, operative mortality is less than 0.5% in PDAs that do not have anomalies and do not have insufficiency or infection.^15^,^16^ This rate is (2.5%) according to a series of 698 cases at John Hopkins hospital. In this study carried out by our clinic, 4 of our patients who underwent PDA elimination with ligation technique were found to have residual PDA between the 2nd and 6th months after discharge, and PDA elimination was then achieved with coil embolization by the pediatric cardiology clinic. One patient was hospitalized in our clinic after detecting residual PDA in routine polyclinic controls 1 year after the operation and the PDA was eliminated with the division 90 method. In one of our patients, a left thoracic hematoma was detected in the 8th postoperative hour.

Increased morbidity associated with PDA may be secondary to surgical or anesthetic complications, side effects of the procedure itself, or postoperative instability. Possible complications related to PDA ligation include surgical and anesthetic complications, postoperative hemodynamic instability, including hypotension and shock, vocal cord paresis, long intubation, and mechanical ventilation. In PDA operations performed at our clinic, mortality was calculated as 8 in 126 patients. Two of our patients who underwent ligation technique with left posterolateral thoracotomy were followed by mechanical ventilator on the 1st postoperative day and one with mechanical ventilator complications died on the 5th postoperative day due to aspiration pneumonia. None of our patients who were operated for an isolated PDA diagnosis died.

In adult patients, CT is very useful for planning the type of surgical approach and to detect calcification of the ductus in elderly individuals presenting with a PDA. The PDA was closed in adult patients who had not developed Eisenmenger syndrome and in patients with suitable pulmonary pressures. The approaches can be via lateral thoracotomy, median sternotomy, or video-assisted thoracoscopic ligation. Successful closure rate is almost 100%, but the morbidity of surgery is higher and the length of stay is longer compared with percutaneous closure.^17^ If the patient has comorbid conditions, such as atherosclerotic aorta and vascular pathologies, open surgery may be preferred for these patients. Today, due to advances in development of new devices, transcatheter PDA closure has gained a special place in the treatment of patients and has become a preferred alternative to surgery. In newborn patients, NSAIDs containing indomethacin and ibuprofen have been used successfully for treatment of PDA and emerging evidence demonstrates their potency as an effective alternative agent to acetaminophen.^18^ Both indomethacin and ibuprofen are non-selective COX inhibitors that work through inhibition of prostaglandin production from free arachidonic acid.^18^,^19^ Acetaminophen has downside effects on the POX site, preventing conversion of PGG2 to PGH2. All three drugs eventually cause PGE2, PGI2, and thromboxane to decrease, and finally the PDA to narrow and close.^20^ Surgical ligation has become the standard treatment for hemodynamically significant treatment, especially in premature babies in whom medical management has been unsuccessful. However, transcatheter PDA closure with smaller devices and improved catheter technology is also possible in young infants.^21^ Since transcatheter PDA closure is among the safest interventional cardiac procedures and is now considered the method of choice for PDA closure in infants above 5 kgs, a variety of devices have been approved for safe and effective PDA closure.^21^ Although the morbidity and mortality attributable to PDAs in premature infants are well established, the ideal management strategy is unknown. It has been shown that early surgical ligation may have a better respiratory result and better nutritional status compared to late surgical ligation.^22^ Today, with technological development and increased experience with transcatheter closure, device closure of PDA has lower risk of complications than surgical ligation.

Our study has limitations inherent to a retrospective analysis with a limited sized sample from a single institution. In addition, our method of determining complications associated with each procedure may differ compared to other institutions and potentially limit the generalizability of our results. In addition, PDA has a complex and multifactorial genetic cause. Evidence suggests that PDA is probably two overlapping disorders; where preterm PDA is due to structural and physiological immaturity and full term PDA is due to genetic changes. PDA is a common finding in dysmorphic syndromes associated with congenital heart disease and an estimated 10% of PDA cases are associated with chromosomal abnormalities.^23^

Although medical management is part of the treatment strategy, the primary approach is surgical intervention. We are in favor of surgical closure of isolated PDAs with a confirmed diagnosis, for those who are symptomatic and for those who are asymptomatic, at pre-school age and preferably at the age of 0-1. Percutaneous PDA closure can be employed in preterm infants and is well tolerated. Closure is indicated in all patients with evidence of LV volume overload and in those with pulmonary hypertension (without evidence of Eisenmenger physiology). However, the technique is currently more expensive than surgical ligation. Non-surgical transcatheter PDA closure is a new approach to permanent PDA in advanced neonates and requires further evaluation. Indications for PDA closure are largely similar in adults and children. Although there are not many differences in terms of mortality among surgical treatment methods, the shorter duration and reliability of ligation surgery compared to division can be accepted as an advantage in favor of ligation with these two methods, which are not different in terms of mortality and morbidity. Adult PDA may need extracorporeal circulation or endovascular treatment with aortic endoprosthesis or percutaneous closure with an Amplatz device. A surgical approach with thoracotomy for PDA closure is a treatment that can be employed successfully in children and adults as well as in older patients. Although surgical mortality and morbidity risks are elevated in PDA, the procedure offers significant therapeutic and prognostic benefits, with excellent postoperative outcomes.

PDA is a complex pathophysiology that results in markedly variable clinical outcomes. To provide more targeted, individualized treatments, studies should be prioritized to better understand the molecular mechanisms of ductal closure, the impact of each patient's clinical and echocardiographic biomarkers on both treatment success and improved outcomes, as well as genetic variants and drug targets in drug metabolism. It is recommended to combine clinical and cardiac imaging parameters that recognize a hierarchical pattern of reverse ductal sequelae, regardless of patient age. The critical need for contemporary, pragmatic clinical trials evaluating the impact of current PDA treatments on important patient outcomes is recognized.

## Data Availability

Research data is available in the body of the article.
